# Effect of Fiber Posts on Stress Distribution of Endodontically Treated Upper Premolars: Finite Element Analysis

**DOI:** 10.3390/nano10091708

**Published:** 2020-08-29

**Authors:** Maciej Zarow, Mirco Vadini, Agnieszka Chojnacka-Brozek, Katarzyna Szczeklik, Grzegorz Milewski, Virginia Biferi, Camillo D’Arcangelo, Francesco De Angelis

**Affiliations:** 1 “NZOZ SPS Dentist” Dental Clinic and Postgraduate Course Centre—pl. Inwalidow 7/5, 30-033 Cracow, Poland; maciej.zarow@dentist.com.pl; 2 Unit of Restorative Dentistry and Endodontics, Department of Medical, Oral and Biotechnological Science, “G. D’Annunzio” University of Chieti—Via dei Vestini 31, 66100 Chieti, Italy; metalfree79@hotmail.com (M.V.); virg.bif@gmail.com (V.B.); cdarcang@unich.it (C.D.); 3 Institute of Applied Mechanics, Cracow University of Technology—Warszawska 24, 31-155 Cracow, Poland; achojnacka@mech.pk.edu.pl (A.C.-B.); milewski@mech.pk.edu.pl (G.M.); 4 Department of Integrated Dentistry, Jagiellonian University Medical College—Montelupich 4, 31-155 Cracow, Poland; k.szczeklik@uj.edu.pl

**Keywords:** fiber post, finite element analysis, maxillary first premolar, MOD nanocomposite restoration, stress distribution

## Abstract

By means of a finite element method (FEM), the present study evaluated the effect of fiber post (FP) placement on the stress distribution occurring in endodontically treated upper first premolars (UFPs) with mesial–occlusal–distal (MOD) nanohybrid composite restorations under subcritical static load. FEM models were created to simulate four different clinical situations involving endodontically treated UFPs with MOD cavities restored with one of the following: composite resin; composite and one FP in the palatal root; composite and one FP in the buccal root; or composite and two FPs. As control, the model of an intact UFP was included. A simulated load of 150 N was applied. Stress distribution was observed on each model surface, on the mid buccal–palatal plane, and on two horizontal planes (at cervical and root-furcation levels); the maximum Von Mises stress values were calculated. All analyses were replicated three times, using the mechanical parameters from three different nanohybrid resin composite restorative materials. In the presence of FPs, the maximum stress values recorded on dentin (in cervical and root-furcation areas) appeared slightly reduced, compared to the endodontically treated tooth restored with no post; in the same areas, the overall Von Mises maps revealed more favorable stress distributions. FPs in maxillary premolars with MOD cavities can lead to a positive redistribution of potentially dangerous stress concentrations away from the cervical and the root-furcation dentin.

## 1. Introduction

Endodontically treated teeth (ETT) are structurally different from non-restored vital teeth. The differences include changes in the amount of the tooth structure, in the properties of dentine, and in proprioception. For these reasons, they require specific restorative treatments [1]. The concepts on the basis of endodontically treated tooth restoration have undergone significant changes over the last years, mainly in the direction of a better preservation of tooth structures and tissues [2,3,4]. The retention of restorative materials, in cases with substantial coronal dentine loss, can be enhanced by an endodontic post with the aim of improving the longevity of the restoration [5,6,7,8]. Cast gold posts and cores have been regarded as the “gold standard” for many years [9]; then, prefabricated titanium posts have emerged as an alternative solution. However, the use of metal posts appeared associated to high fracture indexes, ranging in some studies between 2% and 4% [10], which has been attributed to stress concentration on dentin substrate [11,12]. Non-adhesive posts are not able to homogeneously spread forces along the tooth–post interface area, typically concentrating the stresses in the dentin around the central third of the canal [13].

Bonded restorations, on the other hand, demonstrate a positive influence on tooth mechanical properties [3,4,14] and may guarantee excellent clinical outcomes [15,16]. For this reason, currently, adhesive fiber posts have become popular in dental practice [7,17]. They are made up of pre-stretched fibers of carbon, glass, or quartz soaked in an epoxy-polymer or bisphenol A-glycidyl methacrylate (Bis-GMA) resin matrix [18,19]. They are used mainly because of their advantageous mechanical properties, such as the elastic modulus, which is closer to that of dentine (about 18 GPa). The evidence supports that fiber posts, used in combination with adhesive techniques, allow for the creation of a homogeneous and integrated unit, involving the restorative materials, the dental substrates, and all the interfaces [2,20,21,22,23,24,25,26,27].

Recently, on the other hand, an increasing number of papers have been advocating the use of direct composite restorations without any post for ETT [28,29,30,31,32], stressing the concept that the purpose of a post should be just to retain the core (whenever required) instead of increasing the intrinsic resistance of the root [33,34]; the introduction of innovative and advanced resin-based restorative materials would probably make such a clinical approach even more reliable. Nowadays, in fact, emerging nanotechnologies and improved nanoscale knowledge allows the characterization and control of materials at the atomic or molecular level on a scale of less than 100 nm [35,36]. Composite resins with nanodimensional filler particles are easier to shape and show better polishability [37]. It has been reported that the wear resistance of a resin composite is strongly dependent on the filler load and on the filler particle size, beside the chemical composition of its resin matrix and the bond strength between filler particles and the matrix [38,39]. Cao et al. observed for nanocomposites a better resistance to the abrasive wear [40]. As a result of the reduced abrasive wear and of the subsequently reduced filler plucking, nanocomposites offer better surface smoothness and provide superior gloss retention [41,42], which is a great advantage especially for large direct restorations that require intraoral finishing and polishing procedures. Furthermore, based on some studies, other mechanical properties such as the flexural strength, compressive strength, or diametral tensile strength may be significantly improved on nano-based materials compared to conventional hybrid composites [41,43,44,45]. Due to their enhanced features, nanoparticles-filled materials might oppose a more effective resistance during the propagation of microfractures generated following cyclic fatigue loading [46] and could clinically display improved performance in direct restorations of endodontically treated teeth, even when avoiding the use of a fiber post [47].

The greatest advantage of using only adhesive procedures and direct resin composite restorations is that all the dental substrates remaining after caries removal and root canal treatment can be easily preserved, and it has been clearly shown that the survival rates of endodontically treated teeth are strongly related to the preservation of tooth structure [48,49]. However, this specific research topic seems still partially controversial [6,10,14,50,51,52,53,54,55]. In particular, it is not clear whether or not restorations based on this “simplified” approach could attain the same strength as resin composite restorations placed together with a fiber post. Therefore, the influence of glass fiber post placement on endodontically treated maxillary teeth, especially on premolars (that are particularly prone to fracture), would definitely benefit from further investigations [55,56].

The stress distribution within the root of an endodontically treated tooth (which is typically assessed by means of finite element analysis (FEA) and with Von Mises stress diagrams) may play a key role in the full understanding of the fracture resistance and crack propagation mechanisms along weakened tooth surfaces [57,58,59].

As a consequence, the objective of the present study was to evaluate, using a 3D finite element analysis, the stress distribution in endodontically treated maxillary first premolars (MFPs) presenting mesial–occlusal–distal (MOD) cavities and directly restored by using 3 different commercially available nanohybrid resin composites without any fiber post or associated to the following: 1 fiber post in the palatal root canal; 1 fiber post in the buccal root canal; or 2 fiber posts in both the palatal and buccal root canals. The null hypothesis tested was that the fiber post placement could not significantly affect the maximum stress values and the stress distribution in endodontically treated premolars restored with direct MOD nanohybrid resin composite fillings.

## 2. Materials and Methods

For the purposes of the study, ANSYS v.13 software (ANSYS Inc., Canonsburg, PA, USA) was employed. A plaster cast of an MFP (scale 5:1) was used to determine the external shape of the tooth thanks to the reverse engineering technique, using a Smartech 3D optical measurement system. Almost 15,000 surface points were obtained by using structural white light beam scanning from 10 different directions. The set of collected points were assembled in a 3D surface-frame structure by using a 3D computer-aided design (CAD) Catia system (Dassault Systemes, Vélizy-Villacoublay, France), which enabled increasing the tooth model to actual size. All tooth structures were modeled using isometry, translation, and rotation of the relevant elements of the external surface, and their volumes were determined according to the literature [60]. Then, the outer geometry volume was created to model the periodontal ligaments (approximately 0.20 mm) and the alveolar ridge [60].

The 3D solid model was exported into ANSYS format so as to generate the definitive meshed premolar model (16,125 nodes and 89,727 tetrahedral elements).

By using finite element modeling (FEM), 5 clinical situations were simulated and assigned to 1 control and 4 experimental groups. In the control group (Group 1), the tooth was maintained intact. The other four experimental groups simulated an endodontically treated tooth restored following the subsequent treatment modalities:

Group 2: MOD nanohybrid resin composite restoration;
Group 3: One fiber post in the palatal root canal and MOD nanohybrid resin composite restoration;
Group 4: One fiber post in the buccal root canal and MOD nanohybrid resin composite restoration;
Group 5: Two fiber posts and MOD nanohybrid resin composite restoration.

**Figure 1:**
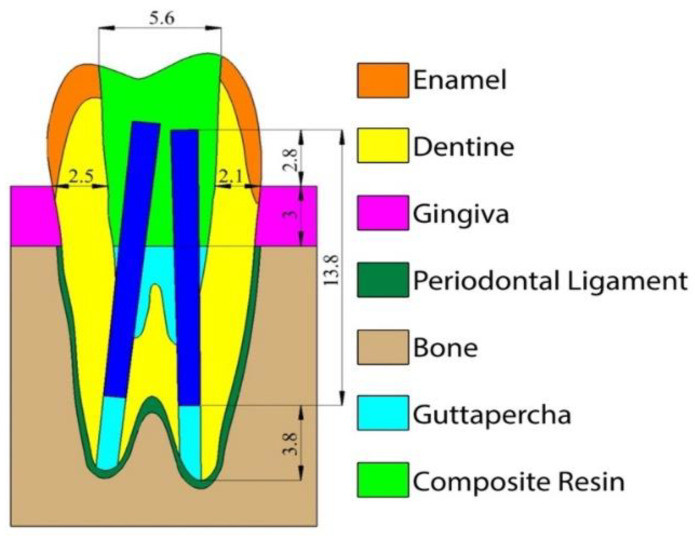
Schematic representation of the cavity configuration and post-space depth used for the simulated models.

The MOD cavities were standardized simulating a severe dental structure loss, with a dentine thickness of 2.1 mm and 2.5 mm remaining respectively on the buccal and on the palatal walls (Figure 1).

Concerning the resin composite material employed for the MOD restoration, all analyses were replicated three times, using the mechanical parameters from three different nanohybrid and commercially available resin composites: Enamel Plus BioFunction (Micerium, Avegno, Italy), Filtek Z350 XT (3M ESPE, Seefeld, Germany), and Grandio (VOCO, Cuxhaven, Germany).

The properties attributed to the isotropic materials included in the model are presented in Table 1 [61,62,63,64,65].

The size of the post used in groups 3, 4, and 5 corresponded to a commercially available fiber post (EnaPost CP0210: Micerium, Avegno, Italy) with 1.0 mm apical diameter and 2% taper. A 3.8 mm guttapercha apical seal was left to mimic standard and acceptable clinical conditions [66]. The orthotropic properties employed for the fiber post have been deduced from previous papers [67] and are listed in Table 2.

A total of 13 models were subjected to the FEA: 1 control (unrestored) model and 12 restored models (4 different restorations for each one of the 3 nanohybrid resin composites investigated). All models were loaded by applying a force of 150 N to the triangular ridges of the buccal and palatal cusps (the force was distributed on the surfaces through the nodes of the mesh elements, simulating the occlusal contacts of the opponent dentition), respectively at an angle of 25 and 22 degrees with the tooth long axis. The angulation of the load vectors resulted from the anatomy and slope of the cusps, with a normal direction of the force in relation to the surfaces of the ridges.

In the 5 different groups (and for each one of the three different restorative materials under investigation), Von Mises stress distributions maps, based on a linear static structural analysis, were calculated on the following reference surfaces:

- on the whole outer model (i.e., tooth) surface;
- along the mid buccal–palatal plane, following the tooth long axis;
- on a cervical horizontal plane, placed at the level of the alveolar ridge;
- on a horizontal plane placed at the level of the root furcation;
- on a cervical horizontal plane (alveolar ridge), taking into account just dental structures;
- on a horizontal plane at the root-furcation level, taking into account just dental structures.

Individually analyzing all the above stress distribution maps, the maximum Von Mises stress values were recorded in every group and for every restorative material.

Subsequently, based on the default legend adopted for the stress/strain descriptions within the employed ANSYS v.13 FEM software, on each stress distributions map, the entire range of stress values observed (included between 0 and the maximum Von Mises stress) was equally segmented out into 7 (discrete and ordinal) stress ranks. Each stress rank was labeled using one of the following 7 colors: dark-blue, blue, light-blue, green, yellow, orange, red (from the lowest to the highest stress intensity). With the aim of allowing a quantitative analysis of the stress distribution, the surface areas corresponding to each different color on the distribution maps were measured using GIMP 2.10.18 software (GIMP Development Team, www.gimp.org), and this provided an objective and quantitative parameter to evaluate the spatial extension of every specific stress rank. Spatial extension data were recorded both as the absolute count of pixels and as a pixel percentage of the whole map surface. Then, data were arranged on crosstabs (with “stress ranks” on rows and “experimental/control groups” on columns), and statistical analyses were performed to assess the effect of the belonging “experimental/control group” on the spatial extension of each particular stress rank (i.e., the surface extension of each corresponding color). Analyses were conducted by using chi-squared tests (with Yates’ correction for 2 × 2 tables) or Fisher’s exact tests (when low expected values occurred). The level of α was set at 0.05 for all tests. R Core Team software, version 3.6.3, was used (R Core Team (2019). R: A language and environment for statistical computing. R Foundation for Statistical Computing, Vienna, Austria. URL https://www.R-project.org/).

## 3. Results

**Figure 2:**
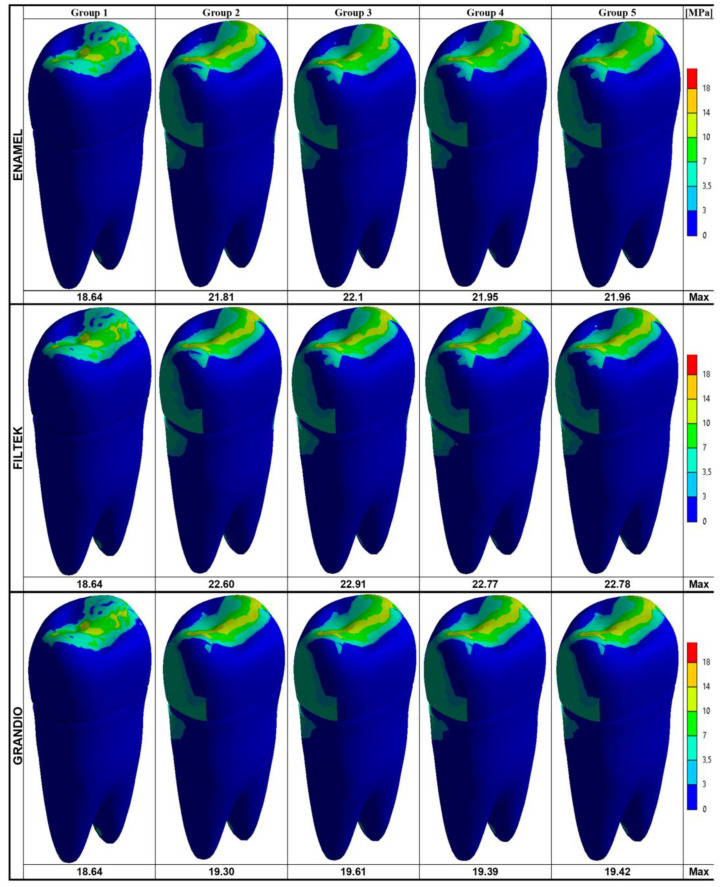
Von Mises maps and Maximum Von Mises stress values (MPa) recorded on the surface of the tooth model. The legend on the right side shows the colors (and the upper and lower limits) corresponding to the seven stress ranks used to analyze the stress distribution.

**Figure 3:**
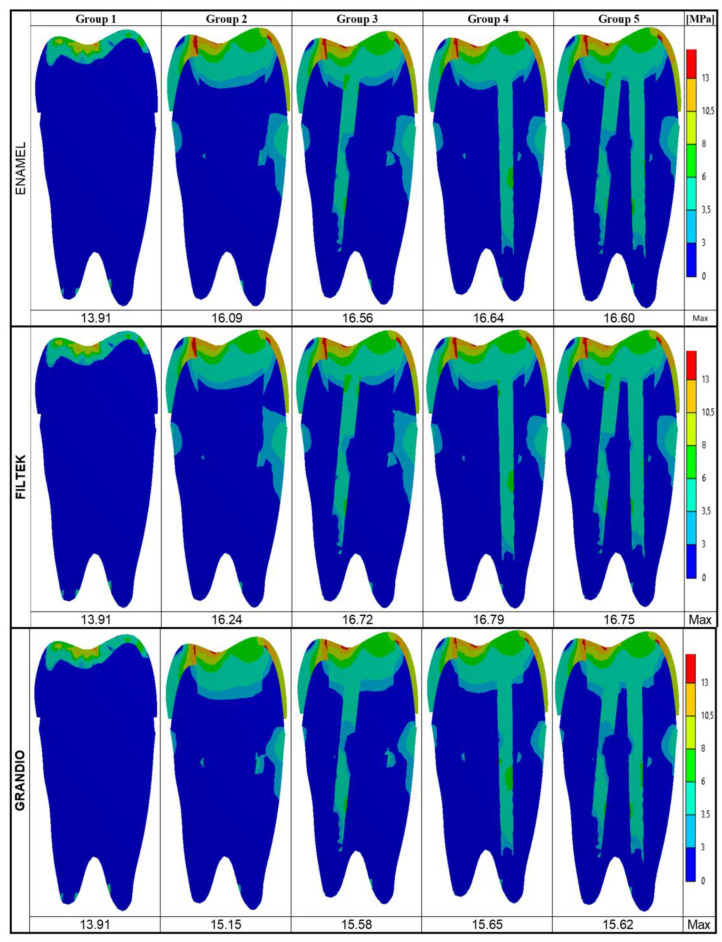
Von Mises maps and Maximum Von Mises stress values (MPa) recorded on the middle buccal–palatal plane. The legend on the right side shows the colors (and the upper and lower limits) corresponding to the seven stress ranks used to analyze the stress distribution.

The maximum Von Mises stresses and the stress distribution maps achieved on the model surfaces and along the buccal–palatal planes are respectively shown in Figure 2 and Figure 3.

**Figure 4:**
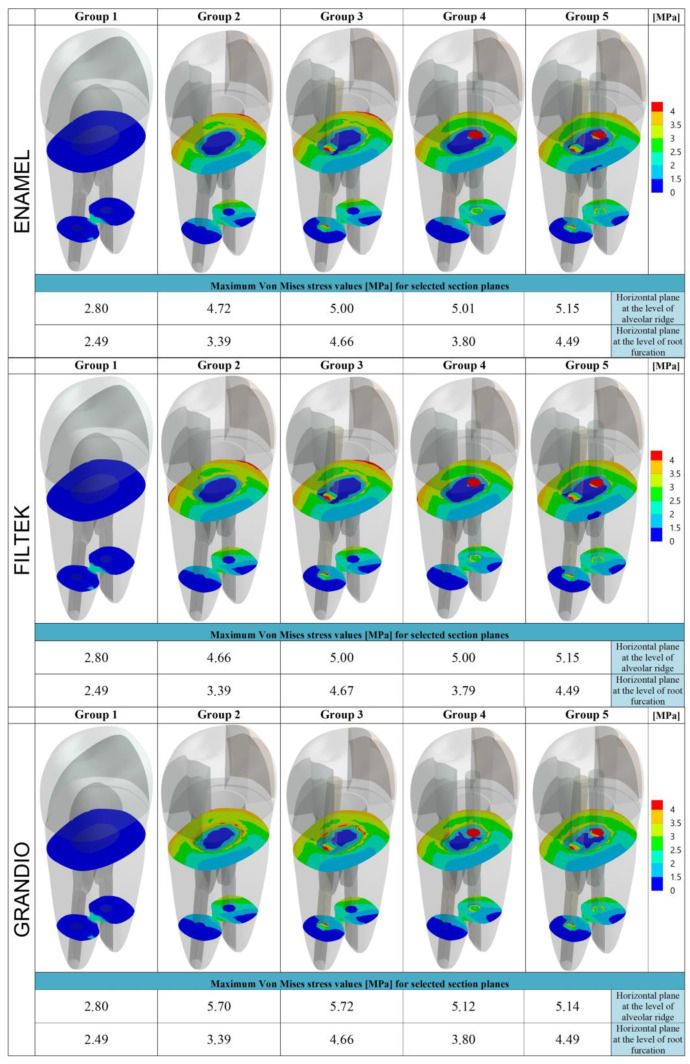
Von Mises maps and Maximum Von Mises stress values (MPa) recorded along the level of the alveolar ridge (cervical horizontal plane) and at the root-furcation level. The legend on the right side shows the colors (and the upper and lower limits) corresponding to the seven stress ranks used to analyze the stress distribution.

In Figure 4, data recorded on two horizontal planes (at the cervical and at the root-furcation level) are summarized.

**Figure 5:**
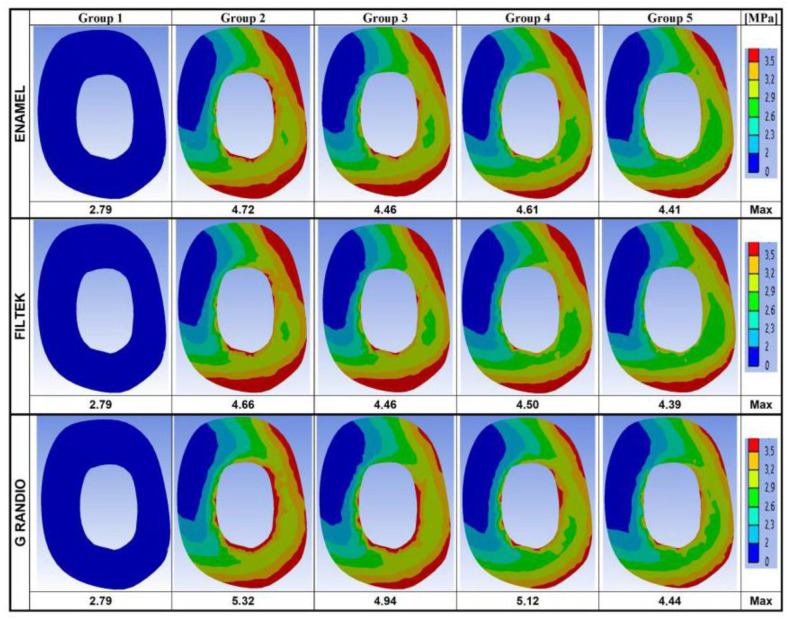
Von Mises maps and Maximum Von Mises stress values (MPa), limited to the radicular dentin, recorded along the level of the alveolar ridge. The legend on the right side shows the colors (and the upper and lower limits) corresponding to the seven stress ranks used to analyze the stress distribution.

**Figure 6:**
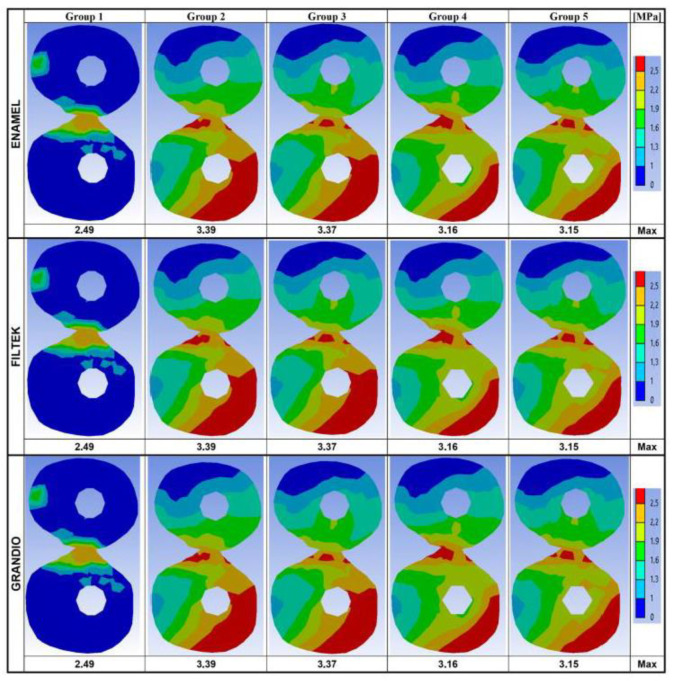
Von Mises maps and Maximum Von Mises stress values (MPa), limited to the radicular dentin, recorded at the root-furcation level. The legend on the right side shows the colors (and the upper and lower limits) corresponding to the seven stress ranks used to analyze the stress distribution.

Maximum stresses and stress distribution maps calculated on the same horizontal planes, but limiting the analysis to the dental tissue, are given in Figure 5 (cervical level) and Figure 6 (root-furcation level).

All the data obtained following the quantitative stress distribution analysis have been provided as Supplementary Material, together with the related results of the performed statistical tests.

In every group, the highest Von Mises stress values were observed on the occlusal aspect of the outer model surface (Figure 2). In the groups where the tooth was not intact (Groups 2–5), the highest stress values ranged between 21.81 and 22.1 MPa (for Enamel Plus BioFunction), 22.60 and 22.91 MPa (for Filtek Z350 XT) and 19.30 and 19.61 MPa (for Grandio) and seemed concentrated at the occlusal interface between the composite and tooth structure. In the intact tooth (Group 1), the maximum Von Mises stress on the outer occlusal surface was slightly reduced (18.64 MPa).

In addition, dealing with the buccal–palatal sections (Figure 3), the maximum Von Mises stress values were observed toward the occlusal surface of each model and seemed concentrated along the composite/tooth interface. On those sections, the highest values were recorded in Group 4 (16.64 MPa for Enamel Plus BioFunction; 16.79 MPa for Filtek Z350 XT; 15.65 MPa for Grandio) and the lowest values were recorded on the intact tooth (13.91 MPa); the maximum Von Mises stress values for restored models (Groups 2–5) were generally quite similar (16.09–16.64 MPa for Enamel Plus BioFunction; 16.24–16.79 for Filtek Z350 XT; 15.15–15.65 for Grandio).

When focusing on the root, the stress distribution maps obtained on the buccal–palatal sections (Figure 3) and on the entire outer tooth surface (Figure 2) of restored teeth (Groups 2–5) revealed that the stress concentration on the external root surface was higher in the cervical regions.

As far as the distance from the occlusal surface (and thus form the load application point) increased, the maximum stresses recorded appeared less intense. Along the cervical–horizontal plane (Figure 4), the lowest maximum Von Mises stress value was observed on the outer surface of the intact tooth (2.80 MPa), while the highest Von Mises stress values were recorded within the body of the fiber post in Group 5, for Enamel Plus BioFunction (5.15 MPa) and Filtek Z350 XT (5.15 MPa), and in Group 3, for Grandio (5.72 MPa). Similarly, along the horizontal sections placed at the root-furcation level (Figure 4), the lowest Von Mises stress value was observed in the intact tooth (2.49 MPa) and the highest stress values were observed inside the fiber post in Group 3 (4.66 MPa for Enamel Plus BioFunction; 4.67 for Filtek Z350 XT; 4.66 for Grandio).

Considering the root-furcation sections (Figure 4), the maximum stress values recorded in Group 2 (restored without fiber posts) were steadily reduced compared to those observed in Groups 3, 4, and 5 (restored with one or two posts). This was true also for the cervical sections, particularly for Enamel Plus BioFunction and Filtek Z350. However, when for all the horizontal sections (cervical and at root furcation) the stress distribution maps were calculated taking into account just the root dentin of the restored models (Figure 5 and Figure 6), with all composites, a reduction in the maximum Von Mises stress values could be observed if groups with fiber posts (Groups 3, 4, and 5) were compared to models restored without any post (Group 2). Moreover, in Figure 5 and Figure 6, the Von Mises stress distribution maps appeared visually more favorable in the presence of fiber posts (Groups 3, 4, and 5), as the spatial extension of the most intense stress ranks (corresponding to surface areas labeled in red, orange and yellow) were considerably reduced (compared to Group 2), and the least intense stress ranks (labeled from green to dark-blue) became dominant. Those differences in the stress distributions were statistically significant for all composite under investigation (*p* < 0.05) (data provided as Supplementary Material) and highlighted a certain stress relief occurring into the dentin on the horizontal sections analyzed, which was most likely consequent to the stress concentration increase within the fiber posts (as observed in Figure 4).

## 4. Discussion

The present 3D numerical analysis focused on endodontically treated and widely restored maxillary first premolars, as they fracture rather frequently [56,68]. Owing to their specific morphology and position in the tooth arch, premolars are subjected to higher masticatory loads than frontal teeth, but they are also more likely than molars to be subjected to lateral forces during mastication [10]. Compared to molars, premolars have less tooth substance and smaller pulp chambers to retain a core build-up after endodontic treatment. Moreover, the preparation of an endodontic access cavity increases the possibility of cusp fractures following the cusp deflection during function [69], which is higher for endodontically treated premolars with MOD preparations [70,71]. In fact, it has been observed how the loss of the marginal walls makes these teeth extremely prone to fracture [10,28,52]. For all the above reasons, premolars might hypothetically require fiber posts more often than molars, especially in the presence of MOD cavities [5,52]. In this study, a large MOD cavity was evaluated with thin residual dentinal walls, since such a severe tooth structure loss is a rather common situation for endodontically treated premolars [72].

According to previous studies [50,61,73], the Von Mises stress values were analyzed on the whole model surface (Figure 2), along the buccal–palatal plane (Figure 3) and along two horizontal planes at the level of the alveolar ridge (cervical) and of the root furcation (Figure 4). Moreover, in the present study, data related to the cervical and root-furcation horizontal planes were further analyzed, limiting the calculation to the root dentinal structures (Figure 5 and Figure 6), in order to better emphasize any stress distribution change occurring in the radicular dentin and maybe preventing/predisposing to catastrophic root fractures.

Based on the present results, irrespective of the particular nanohybrid restorative material employed, the Von Mises stress concentration was highest in the occlusal areas of all the tested groups, which is not surprising as the load was applied occlusally. Interestingly, the highest stresses were recorded at the occlusal interface between the composite restoration and the tooth structure (Figure 2). Even on buccal–palatal sections (Figure 3), the highest Von Mises stresses seemed concentrated along the first occlusal millimeters of the tooth restoration interface. Those findings might indicate the tooth restoration interface as a critical area for fractures that starts form the coronal portion of the tooth. In association with all the nanocomposite materials herein investigated, the use of fiber posts did not reduce the maximum Von Mises stresses on the outer occlusal interface between the tooth and MOD restorations. Probably, in such situations, a more effective way of reducing such a dangerous stress concentration should involve ensuring a full cusp coverage, even using partial adhesive restorations [74,75]. In fact, as shown by Mondelli et al., endodontically treated premolars with MOD cavity preparation and restored with cusp coverage increased their fracture toughness when compared with premolars restored without any cusp protection [76]. This is particularly evident when the residual wall thickness of extensive MOD cavities is less than 2 mm and only cusp coverage (with or without a fiber post) may provide satisfactory fracture resistance [22]. In these cases, an indirect approach is definitely more appropriate due to the extraoral handling of the material, which enhances the extent of polymerization (by controlling light exposure, temperature, humidity, pressure, and time) and improves contours, proximal contacts, occlusal anatomy, and cavo-surface adaptation. However, in the present study, the residual wall thickness was maintained above the traditional 2 mm limit (2.1 mm were left on the buccal and 2.5 mm were left on the palatal wall), which allowed mimicking a clinical scenario of inlay (i.e., without cusp cupping) and direct composite restoration.

Despite the overall maximum stresses that were steadily observed toward the occlusal side of the model, in the present study, particular attention has been paid to the intensity and distribution of stresses accumulated at the cervical and root-furcation levels, as previous FEM studies have already shown how stresses in those areas (where the root dentin thicknesses may be naturally reduced, particularly in upper premolars [77]) can become relatively high and could potentially lead a weakened root to fracture [61,78]. Accordingly, in the present study, neglecting the coronal portion of the model, the stress concentration on the external root surface appeared highest in the cervical regions for every group (Figure 2 and Figure 3).

Although the maximum Von Mises stress values recorded on the horizontal cervical sections did not undergo any promising reduction following fiber post placement, the Von Mises maps suggested that the stress distribution within the dentin was more favorable when fiber posts were placed. In fact, in the groups with fiber posts (Groups 3–5), the most intense stress ranks (labeled in red in Figure 4) were concentrated within the post areas, with a concomitant relief of the highest stresses away from the cervical dentin: this phenomenon was particularly evident when using Enamel Plus BioFunction and Filtek Z350 XT in both Group 4 (one fiber post in the buccal root canal) and Group 5 (two fiber posts). The simulations based on the stress distribution just within the root dentin (Figure 5 and Figure 6) have clearly underlined such a “stress-relieving” effect of the fiber posts. In fact, the surface areas corresponding to the less intense stress ranks in the cervical and root-furcation sections (green and blue in Figure 5 and Figure 6) got gradually and significantly more extended, moving from Group 2 (restoration with no post) to Groups 3–4 (restoration with one post) and to Group 5 (restoration with 2 posts).

The theory of a positive stress redistribution away from the cervical radicular dentin, when fiber posts are used, seems in line with the results of a previous in vitro study by Sorrentino, who demonstrated that endodontically treated premolars with MOD restorations and fiber posts generally underwent restorable failures, while samples restored without posts showed a predominance of non-restorable, subgingival fractures [10]. Similarly, Sulaiman et al. observed that a fiber post in the palatal root would help maintain the restorability in case of fractured endodontically treated maxillary premolars [6]. In particular, fiber posts seemed to perform a protective role toward root fractures, especially for premolar and anterior teeth [14]. On these bases, Zarow et al. recommended the use of fiber posts in anterior teeth and premolars with significantly compromised tooth structure (<50%) [55].

As already mentioned, on the other hand, it should not be neglected that such a research topic is still somehow controversial. A previous and analogous FEM study on endodontically treated maxillary second premolars suggested that stress concentrations at the cervical level could predominate regardless of whether a post is present or not [50]. Fokkinga et al. found no differences between the mean failure load and the failure mode of endodontically treated premolars restored with or without posts [53], and several comparative in vitro studies showed that the use of posts did not significantly increase the fracture resistance [54].

However, it must be underlined how all the conclusions drawn out of in vitro studies need to be backed up with controlled clinical trials before they can be used as recommendations for routine clinical work. From this point of view, clinical studies by Ferrari et al. and Fei at al. have already shown, following an observation period of more than two years, that fiber post placement can lead to a significantly reduced failure risk for endodontically treated premolars [51,52]. Thus, within the limits of a simulated FEM analysis, the relevance of the present results is that they provide a convincing argument supporting all those findings that encourage the use of adhesive fiber posts, as in maxillary first premolars with extensive MOD cavities, they seemed to positively redistribute a certain amount of potentially dangerous stresses far away from the radicular dentin and concentrate them within the fiber post itself. This would not necessarily avoid a mechanical failure, but, maybe, it would hypothetically help making such a failure less catastrophic.

In order to simulate a clinically relevant scenario, in the present study, an occlusal load of 150 N was applied in a way that the load vectors resulted perpendicular to the triangular ridges of the buccal and palatal cusps, thus reproducing the occlusal contacts of the opposing dentition. This particular load intensity was selected as it approximately corresponds to one-half of the maximum values of the biting forces reported in adults for premolars, which seem to widely range in the literature between 200 and 440 N [79]. Several previous laboratory studies have already employed occlusal loads significantly lower than the maximal ones recorded during biting/chewing [50,78,80]: a similar strategy was also herein adopted, as the aim of this research was to observe the stress distribution maps in subcritical conditions [50,78]. Laboratory tests, performed on real extracted human teeth, are important means for gathering useful information about fracture resistance and the mechanical behavior of tooth structures. However, they are generally based on destructive mechanical experiments and have limited capacity to investigate the internal behavior of the structures studied and to clarify the stress–strain relationship in the tooth restoration complex [81]. Indeed, during mastication and function, teeth may be subjected to several changing forces that might modify their direction, point of application, and intensity [82,83]. However, as for many other studies carried out by means of the finite element method, the present research focused on a static loading model, where the load is applied with constant direction and point of application [84,85,86].

In the present FEA, one single model for the first upper premolar was used, in order not to complicate a study design that was already based on two independent variables (type of MOD restoration and resin composite). Nevertheless, it should not be neglected that particular anatomical features of different first maxillary premolars might potentially affect the observed results. Variations may include the general tooth dimensions, the number of roots, the number and configuration of the root canals, and the frequency of their occurrence. The possible influence of such anatomic variability on the maximum stresses and on the stress distribution of differently restored upper premolars could represent an interesting subject for further FEA investigations.

## 5. Conclusions

- When endodontically treated upper premolars with MOD nanohybrid resin composite restorations were subjected to subcritical occlusal loads, the maximum Von Mises stresses seemed concentrated at the occlusal interfaces between the tooth and composite, which indicates those areas to be critical for fractures originating from the coronal portion of the tooth.
- The use of adhesive fiber posts was neither able to reduce the maximum Von Mises stresses recorded on the occlusal surface, nor to optimize the stress distribution in the same areas.
- Concerning the root dentin, on the other hand, when fiber posts were placed, the Von Mises maps revealed a more favorable stress distribution, which could play a positive role in preventing root fractures.
- When fiber posts were present, both on cervical and on root-furcation horizontal sections, the highest levels of stress seemed to be concentrated within the fiber posts and away from the radicular dentin.

## Figures and Tables

**Table 1:** Mechanical properties used for the isotropic materials included in the experimental model.

Material	Modulus of Elasticity (MPa)	Poisson Ratio
Enamel	84,100	0.33
Dentin	18,600	0.31
Gingiva	19.6	0.30
Periodontal ligament	67	0.47
Bone	14,000	0.30
Gutta-percha	69	0.45
Pulp	2	0.45
Enamel Plus BioFunction (nanocomposite resin)	14,000	0.30
Filtek Z350 XT (nanocomposite resin)	12,770	0.31
Grandio (nanocomposite resin)	19,780	0.31

**Table 2:** Material constants of orthotropic fiber post.

Modulus of Elasticity (MPa)			Shear Modulus (MPa)			Poisson’s Ratio		
**E_x_**	**E_y_**	**E_z_**	**G_xy_**	**G_xz_**	**G_yz_**	**υ_xy_**	**υ_xz_**	**υ_yz_**
9500	37,000	9500	3100	3100	3500	0.27	0.27	0.34

## Supplementary Materials

The following are available online at https://www.mdpi.com/2079-4991/10/9/1708/s1, Table S1: Quantitative analysis data of the stress distributions on the mid buccal-palatal plane maps, along the tooth long axis, Table S2: Quantitative analysis data of the stress distributions on cervical horizontal plane maps, placed at the level of the alveolar ridge, Table S3: Quantitative analysis data of the stress distributions on root-furcation horizontal plane maps, Table S4: Quantitative analysis data of the stress distributions on cervical horizontal plane maps, placed at the level of the alveolar ridge, and calculated taking into account just dental tissues, Table S5: Quantitative analysis data of the stress distributions on root-furcation horizontal plane maps, calculated taking into account just dental tissues.
