# Supplementary material for: Effect of Fiber Posts on Stress Distribution of Endodontically Treated Upper Premolars: Finite Element Analysis
Source: Nanomaterials (Basel). 2020 Aug 29;10(9):1708. doi: 10.3390/nano10091708 (PMC7559636; doi:10.3390/nano10091708)

SUPPLEMENTARY DATA

The following supplementary data provide a quantitative analysis of the stress distributions calculated on the different stress distribution maps (see the main text for a detailed description). On each table, data have been arranged on the basis of the stress-rank (rows) and of the experimental/control Group (columns) and the numbers indicate the surface extension of each stress-rank, given as the absolute count of pixels on each map digital image (and as the pixel percentage of the whole map surface). Statistical analyses were performed by using chi-squared tests (with Yates' correction for 2x2 tabels) or Fisher's exact tests (when low expected values occurred) in order to assess the effect of the belonging "experimental/control group" on the spatial extension of each particular stress-rank. The level of α was set at 0.05 and the results of the statistical tests have been indicated in the tables. The surface extensions of each stress-rank (in each different group) have been also graphically summarized in bar charts.
Data are presented considering separately each different restorative material.

1 Data for the mid buccal-palatal plane maps, along the tooth long axis.

1.1 MATERIAL: Enamel Plus Biofunction (Micerium, Avegno, Italy)

Stress-rank	Group					p
	Group 1	Group 2	Group 3	Group 4	Group 5	
0-3 MPa	135830 (93.36%)	106629 (73.56%)	97129 (67.52%)	96465 (67.11%)	87785 (62.92%)	p<0.001
3-3.5 MPa	1034 (0.71%)	11229 (7.75%)	11618 (8.08%)	8325 (5.79%)	8235 (5.90%)	
3.5-6 MPa	5291 (3.64%)	15662 (10.80%)	22977 (15.97%)	26923 (18.73%)	31246 (22.40%)	
6-8 MPa	1896 (1.30%)	5297 (3.65%)	5956 (4.14%)	5977 (4.16%)	6071 (4.35%)	
8-10.5 MPa	1271 (0.87%)	3277 (2.26%)	3341 (2.32%)	3316 (2.31%)	3375 (2.42%)	
10.5-13 MPa	166 (0.11%)	2435 (1.68%)	2374 (1.65%)	2284 (1.59%)	2369 (1.70%)	
13+ MPa	5 (0.00%)	429 (0.30%)	454 (0.32%)	441 (0.31%)	429 (0.31%)	

p - chi-squared test

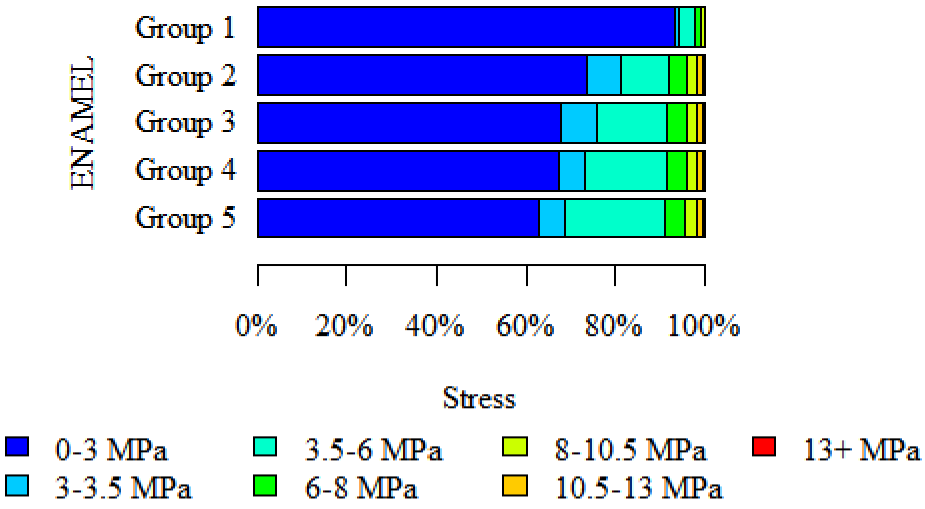

1.2 MATERIAL: Filtek Z350 XT (3M ESPE, Seefeld, Germany)

**Stress**	**Group**					**p**
	**Group 1**	**Group 2**	**Group 3**	**Group 4**	**Group 5**	
0-3 MPa	125307 (92.92%)	109298 (72.68%)	94958 (66.24%)	94372 (66.23%)	86952 (60.47%)	p<0.001
3-3.5 MPa	1010 (0.75%)	12981 (8.63%)	12908 (9.00%)	8851 (6.21%)	10738 (7.47%)	
3.5-6 MPa	5223 (3.87%)	16515 (10.98%)	23190 (16.18%)	27215 (19.10%)	33835 (23.53%)	
6-8 MPa	1878 (1.39%)	5328 (3.54%)	6166 (4.30%)	6048 (4.24%)	6141 (4.27%)	
8-10.5 MPa	1270 (0.94%)	3224 (2.14%)	3195 (2.23%)	3190 (2.24%)	3258 (2.27%)	
10.5-13 MPa	166 (0.12%)	2557 (1.70%)	2447 (1.71%)	2331 (1.64%)	2396 (1.67%)	
13+ MPa	5 (0.00%)	481 (0.32%)	495 (0.35%)	487 (0.34%)	470 (0.33%)	

p - chi-squared test

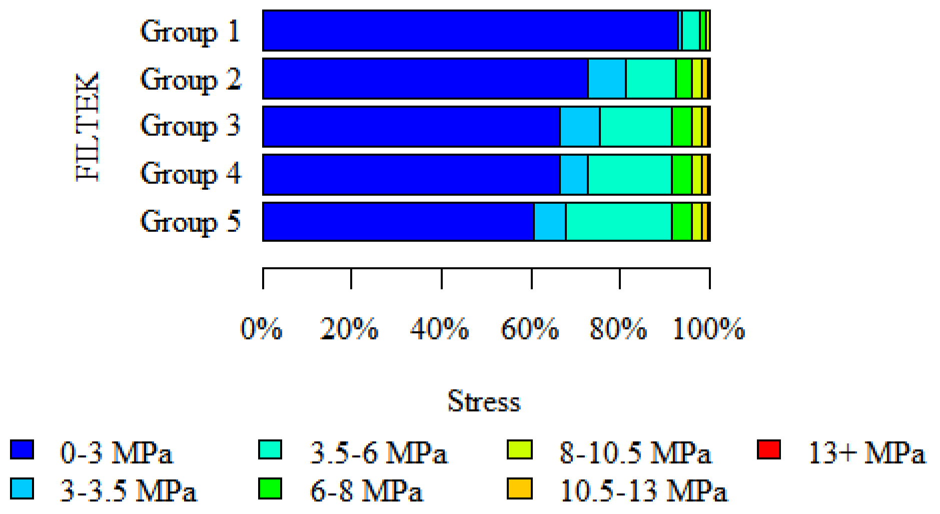

1.3 MATERIAL: Grandio (VOCO, Cuxhaven, Germany)

**Stress**	**Group**					**p**
	**Group 1**	**Group 2**	**Group 3**	**Group 4**	**Group 5**	
0-3 MPa	125307 (92.89%)	106699 (74.89%)	100015 (69.92%)	99039 (68.89%)	244 (0.17%)	p<0.001
3-3.5 MPa	1022 (0.76%)	9388 (6.59%)	10328 (7.22%)	6449 (4.49%)	2164 (1.51%)	
3.5-6 MPa	5246 (3.89%)	14762 (10.36%)	20702 (14.47%)	25565 (17.78%)	3797 (2.64%)	
6-8 MPa	1887 (1.40%)	5583 (3.92%)	5840 (4.08%)	6666 (4.64%)	6256 (4.35%)	
8-10.5 MPa	1270 (0.94%)	3696 (2.59%)	3785 (2.65%)	3707 (2.58%)	31556 (21.96%)	
10.5-13 MPa	166 (0.12%)	2154 (1.51%)	2107 (1.47%)	2066 (1.44%)	8860 (6.16%)	
13+ MPa	5 (0.00%)	199 (0.14%)	263 (0.18%)	263 (0.18%)	90842 (63.21%)	

p - chi-squared test

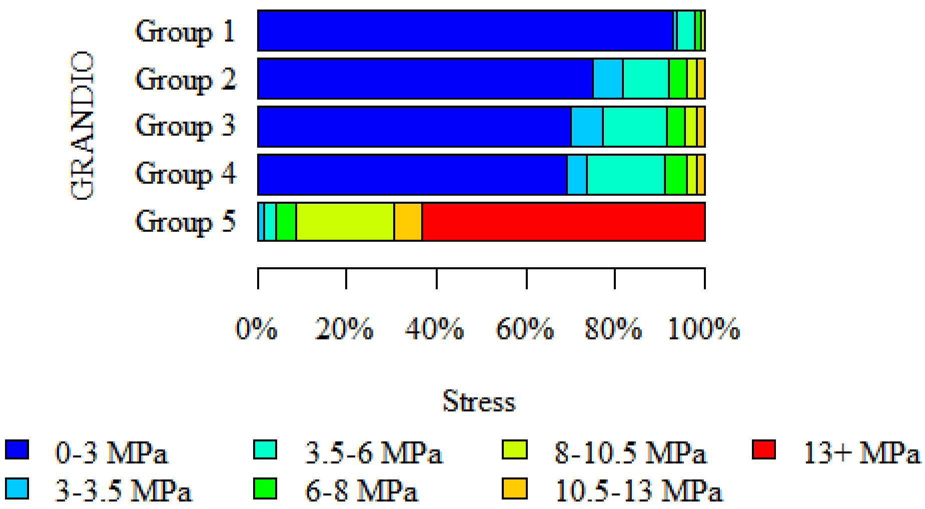

2 Data for cervical horizontal plane maps, placed at the level of the alveolar ridge.

2.1 MATERIAL: Enamel Plus Biofunction (Micerium, Avegno, Italy)

Stress	Group					p
	Group 1	Group 2	Group 3	Group 4	Group 5	
0-1.5 MPa	206967 (99.88%)	25930 (12.47%)	20175 (9.36%)	23736 (11.21%)	20604 (8.93%)	p<0.001
1.5-2 MPa	211 (0.10%)	46677 (22.45%)	52813 (24.49%)	45714 (21.59%)	56441 (24.45%)	
2-2.5 MPa	0 (0.00%)	26159 (12.58%)	32601 (15.12%)	33693 (15.91%)	35659 (15.45%)	
2.5-3 MPa	32 (0.02%)	30788 (14.81%)	37171 (17.24%)	44706 (21.11%)	55430 (24.01%)	
3-3.5 MPa	0 (0.00%)	58643 (28.21%)	52525 (24.36%)	45136 (21.32%)	42660 (18.48%)	
3.5-4 MPa	0 (0.00%)	18691 (8.99%)	17977 (8.34%)	14277 (6.74%)	15231 (6.60%)	
4+ MPa	0 (0.00%)	1022 (0.49%)	2389 (1.11%)	4480 (2.12%)	4793 (2.08%)	

p - chi-squared test

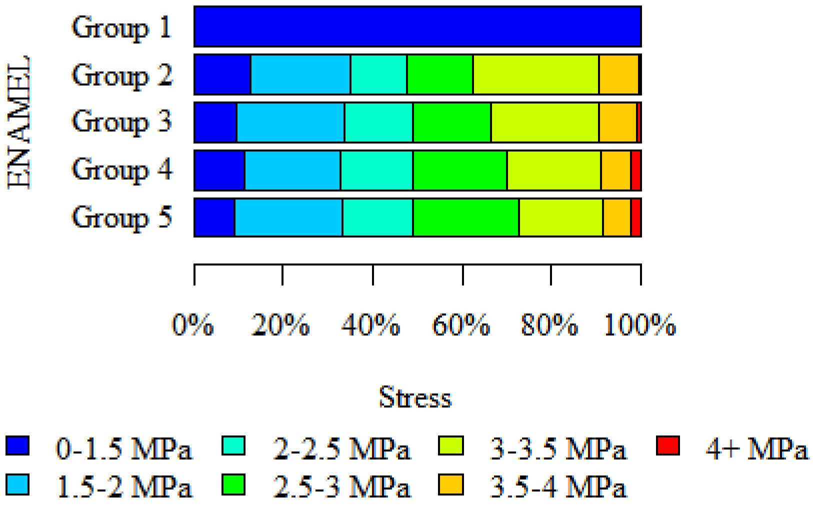

2.2 MATERIAL: Filtek Z350 XT (3M ESPE, Seefeld, Germany)

Stress	Group					p
	Group 1	Group 2	Group 3	Group 4	Group 5	
0-1.5 MPa	206975 (99.88%)	28082 (13.56%)	22866 (11.04%)	25909 (12.50%)	24334 (11.74%)	p<0.001
1.5-2 MPa	213 (0.10%)	38451 (18.57%)	45669 (22.05%)	45052 (21.74%)	46662 (22.52%)	
2-2.5 MPa	21 (0.01%)	32946 (15.91%)	29687 (14.33%)	30145 (14.55%)	29992 (14.47%)	
2.5-3 MPa	10 (0.00%)	29420 (14.21%)	36600 (17.67%)	42951 (20.73%)	49240 (23.76%)	
3-3.5 MPa	0 (0.00%)	54005 (26.08%)	50396 (24.33%)	42851 (20.68%)	36863 (17.79%)	
3.5-4 MPa	0 (0.00%)	21660 (10.46%)	18815 (9.08%)	15628 (7.54%)	15728 (7.59%)	
4+ MPa	0 (0.00%)	2491 (1.20%)	3096 (1.49%)	4658 (2.25%)	4394 (2.12%)	

p - chi-squared test

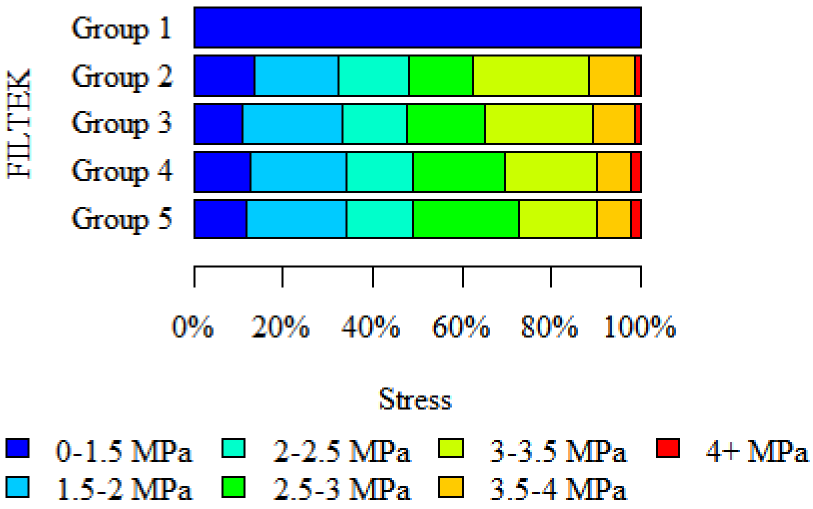

2.3 MATERIAL: Grandio (VOCO, Cuxhaven, Germany)

Stress	Group					p
	**Group 1**	**Group 2**	**Group 3**	**Group 4**	**Group 5**	
0-1.5 MPa	206975 (99.90%)	18556 (8.96%)	13420 (6.35%)	12636 (6.10%)	11247 (4.70%)	p<0.001
1.5-2 MPa	211 (0.10%)	36337 (17.54%)	44424 (21.02%)	45015 (21.73%)	56129 (23.45%)	
2-2.5 MPa	0 (0.00%)	37551 (18.12%)	38660 (18.30%)	40754 (19.67%)	49253 (20.58%)	
2.5-3 MPa	0 (0.00%)	34482 (16.64%)	41235 (19.51%)	46379 (22.38%)	59582 (24.89%)	
3-3.5 MPa	0 (0.00%)	59678 (28.80%)	55600 (26.31%)	49054 (23.68%)	49514 (20.69%)	
3.5-4 MPa	0 (0.00%)	19895 (9.60%)	15784 (7.47%)	8817 (4.26%)	9138 (3.82%)	
4+ MPa	0 (0.00%)	696 (0.34%)	2177 (1.03%)	4538 (2.19%)	4478 (1.87%)	

p - chi-squared test

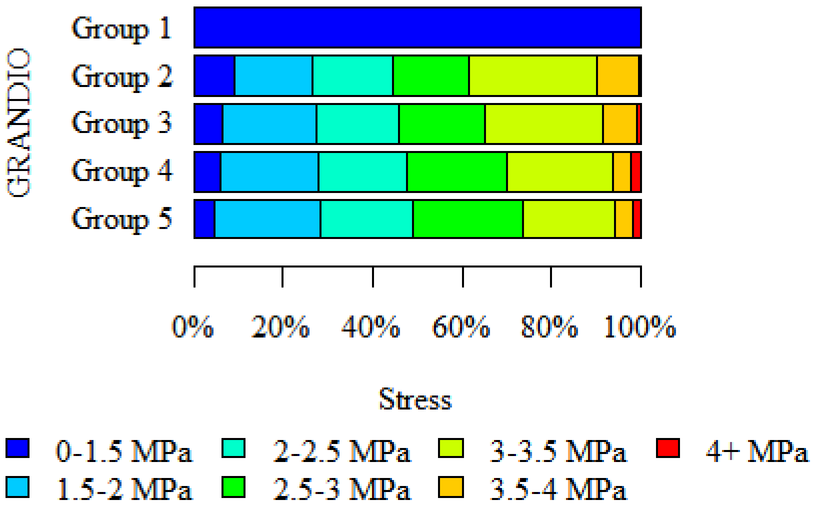

3 Data for root-furcation horizontal plane maps.

3.1 MATERIAL: Enamel Plus Biofunction (Micerium, Avegno, Italy)

Stress	Group					p
	Group 1	Group 2	Group 3	Group 4	Group 5	
0-1 MPa	132596 (85.30%)	22730 (17.73%)	22240 (14.63%)	19680 (14.15%)	20013 (12.00%)	p<0.001
1-1.3 MPa	8783 (5.65%)	16570 (12.92%)	14705 (9.68%)	17573 (12.63%)	17199 (10.31%)	
1.3-1.6 MPa	4760 (3.06%)	23108 (18.02%)	35933 (23.65%)	22466 (16.15%)	30864 (18.50%)	
1.6-1.9 MPa	3090 (1.99%)	20788 (16.21%)	22700 (14.94%)	24979 (17.95%)	28789 (17.26%)	
1.9-2.2 MPa	2838 (1.83%)	12062 (9.41%)	13258 (8.72%)	24694 (17.75%)	29556 (17.72%)	
2.2-2.5 MPa	3375 (2.17%)	14488 (11.30%)	19175 (12.62%)	12425 (8.93%)	18804 (11.27%)	
2.5+ MPa	0 (0.00%)	18484 (14.41%)	23954 (15.76%)	17304 (12.44%)	21580 (12.94%)	

p - chi-squared test

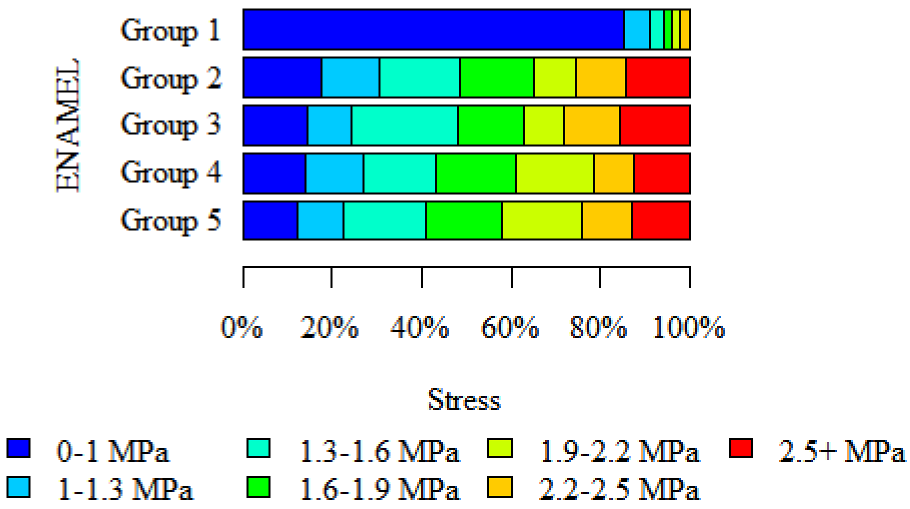

3.2 *MATERIAL: Filtek Z350 XT (3M ESPE, Seefeld, Germany)*

Stress	Group					p
	Group 1	Group 2	Group 3	Group 4	Group 5	
0-1 MPa	131843 (85.07%)	23842 (17.65%)	23173 (14.94%)	22131 (14.37%)	18343 (11.73%)	p<0.001
1-1.3 MPa	9539 (6.15%)	16163 (11.96%)	16581 (10.69%)	18639 (12.10%)	15721 (10.05%)	
1.3-1.6 MPa	4506 (2.91%)	23734 (17.57%)	34670 (22.35%)	24341 (15.81%)	28887 (18.47%)	
1.6-1.9 MPa	3295 (2.13%)	22413 (16.59%)	23425 (15.10%)	28342 (18.40%)	26382 (16.87%)	
1.9-2.2 MPa	2916 (1.88%)	13457 (9.96%)	13338 (8.60%)	26932 (17.49%)	27910 (17.84%)	
2.2-2.5 MPa	2888 (1.86%)	15934 (11.79%)	19397 (12.51%)	13043 (8.47%)	18978 (12.13%)	
2.5+ MPa	0 (0.00%)	19562 (14.48%)	24527 (15.81%)	20570 (13.36%)	20199 (12.91%)	

p - chi-squared test

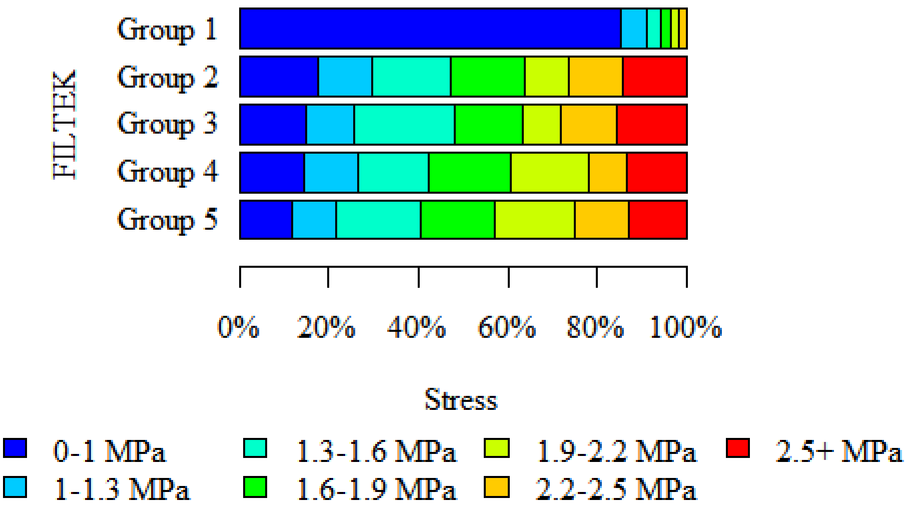

3.3 *MATERIAL: Grandio (VOCO, Cuxhaven, Germany)*

Stress	Group					p
	**Group 1**	**Group 2**	**Group 3**	**Group 4**	**Group 5**	
0-1 MPa	131776 (85.00%)	27639 (21.24%)	23365 (14.65%)	22295 (14.37%)	18628 (11.96%)	p<0.001
1-1.3 MPa	9834 (6.34%)	18888 (14.51%)	18101 (11.35%)	19335 (12.47%)	16386 (10.52%)	
1.3-1.6 MPa	4120 (2.66%)	2741 (2.11%)	33860 (21.23%)	23456 (15.12%)	28053 (18.01%)	
1.6-1.9 MPa	3226 (2.08%)	25119 (19.30%)	24079 (15.10%)	36251 (23.37%)	26410 (16.96%)	
1.9-2.2 MPa	2835 (1.83%)	15458 (11.88%)	14780 (9.27%)	21061 (13.58%)	26850 (17.24%)	
2.2-2.5 MPa	3233 (2.09%)	17938 (13.78%)	20181 (12.65%)	13099 (8.44%)	19038 (12.22%)	
2.5+ MPa	0 (0.00%)	22357 (17.18%)	25110 (15.75%)	19615 (12.65%)	20378 (13.08%)	

p - chi-squared test

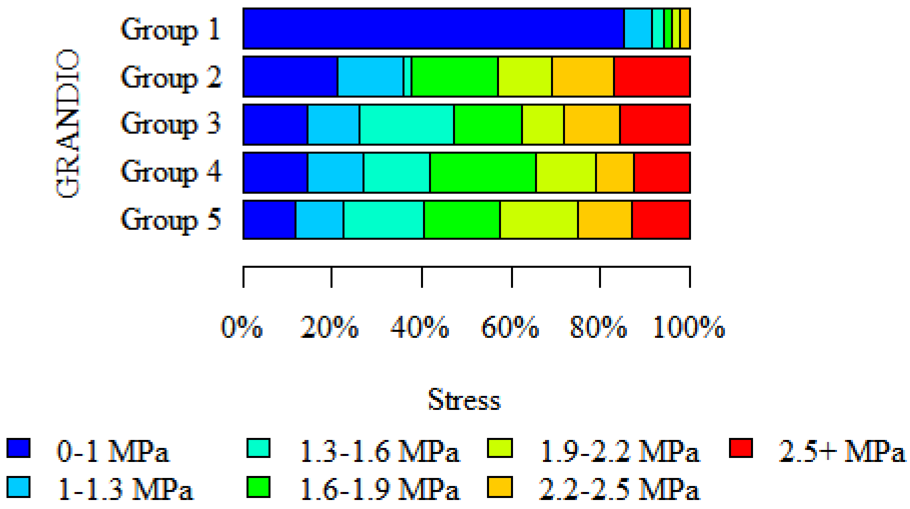

4 Data for cervical horizontal plane maps, placed at the level of the alveolar ridge, and calculated taking into account just dental tissues.

4.1 MATERIAL: Enamel Plus Biofunction (Micerium, Avegno, Italy)

Stress	Group					p
	Group 1	Group 2	Group 3	Group 4	Group 5	
0-2 MPa	240809 (99.97%)	27781 (16.86%)	19145 (19.65%)	29220 (19.80%)	31849 (21.77%)	p<0.001
2-2.3 MPa	29 (0.01%)	17584 (10.67%)	9935 (10.19%)	16841 (11.41%)	16099 (11.00%)	
2.3-2.6 MPa	39 (0.02%)	16791 (10.19%)	9510 (9.76%)	14289 (9.68%)	14873 (10.17%)	
2.6-2.9 MPa	2 (0.00%)	14710 (8.93%)	11056 (11.34%)	22990 (15.58%)	27511 (18.80%)	
2.9-3.2 MPa	0 (0.00%)	35191 (21.36%)	22166 (22.75%)	31888 (21.61%)	28913 (19.76%)	
3.2-3.5 MPa	0 (0.00%)	29536 (17.92%)	14776 (15.16%)	19774 (13.40%)	16260 (11.11%)	
3.5+ MPa	0 (0.00%)	23189 (14.07%)	10866 (11.15%)	12541 (8.50%)	10807 (7.39%)	

p - chi-squared test

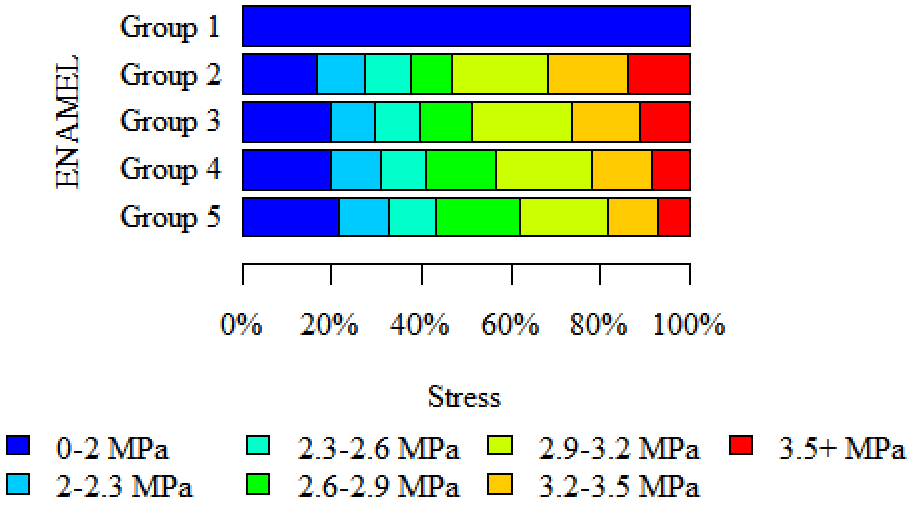

4.2 *MATERIAL: Filtek Z350 XT (3M ESPE, Seefeld, Germany)*

Stress	Group					p
	Group 1	Group 2	Group 3	Group 4	Group 5	
0-2 MPa	240809 (99.97%)	36179 (17.16%)	43177 (19.78%)	45063 (19.87%)	46295 (21.96%)	p<0.001
2-2.3 MPa	29 (0.01%)	21610 (10.25%)	21426 (9.82%)	25384 (11.20%)	21994 (10.43%)	
2.3-2.6 MPa	39 (0.02%)	20783 (9.86%)	20495 (9.39%)	21175 (9.34%)	20485 (9.72%)	
2.6-2.9 MPa	2 (0.00%)	18868 (8.95%)	25468 (11.67%)	36506 (16.10%)	40567 (19.24%)	
2.9-3.2 MPa	0 (0.00%)	44711 (21.21%)	49269 (22.57%)	47184 (20.81%)	40939 (19.42%)	
3.2-3.5 MPa	0 (0.00%)	37597 (17.84%)	31673 (14.51%)	29343 (12.94%)	22167 (10.51%)	
3.5+ MPa	0 (0.00%)	31033 (14.72%)	26747 (12.25%)	22087 (9.74%)	18369 (8.71%)	

p - chi-squared test

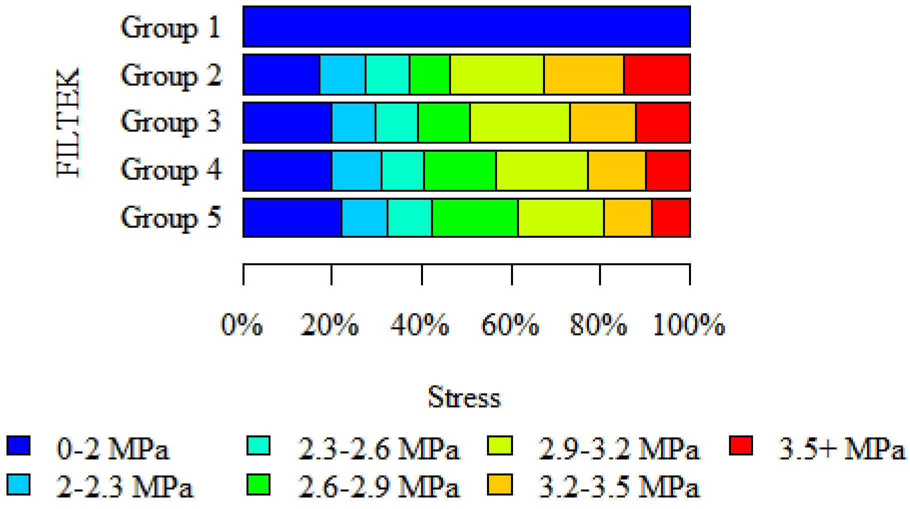

4.3 MATERIAL: Grandio (VOCO, Cuxhaven, Germany)

Stress	Group					p
	Group 1	Group 2	Group 3	Group 4	Group 5	
0-2 MPa	240809 (99.97%)	34370 (16.63%)	41300 (19.74%)	44457 (20.01%)	45543 (22.00%)	p<0.001
2-2.3 MPa	29 (0.01%)	24838 (12.02%)	23526 (11.24%)	28390 (12.78%)	24597 (11.88%)	
2.3-2.6 MPa	39 (0.02%)	22278 (10.78%)	22856 (10.92%)	24079 (10.84%)	23931 (11.56%)	
2.6-2.9 MPa	2 (0.00%)	20732 (10.03%)	22746 (10.87%)	31706 (14.27%)	35092 (16.95%)	
2.9-3.2 MPa	0 (0.00%)	41651 (20.16%)	47574 (22.73%)	49298 (22.19%)	43565 (21.04%)	
3.2-3.5 MPa	0 (0.00%)	38399 (18.58%)	33889 (16.19%)	33078 (14.89%)	26961 (13.02%)	
3.5+ MPa	0 (0.00%)	24385 (11.80%)	17381 (8.31%)	11160 (5.02%)	7351 (3.55%)	

p - chi-squared test

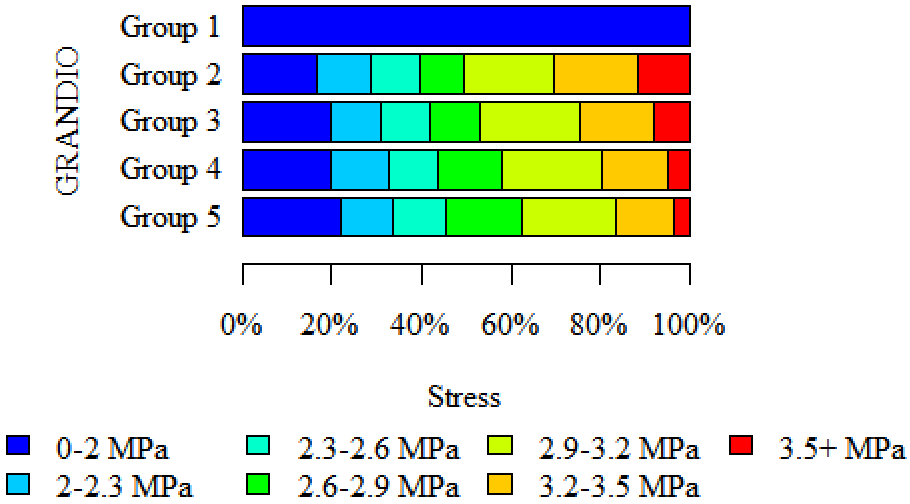

5 Data for root-furcation horizontal plane maps, calculated taking into account just dental tissues.

5.1 MATERIAL: Enamel Plus Biofunction (Micerium, Avegno, Italy)

Stress	Group					p
	**Group 1**	**Group 2**	**Group 3**	**Group 4**	**Group 5**	
0-1 MPa	188510 (84.15%)	17771 (10.70%)	16663 (10.98%)	17469 (11.27%)	18560 (12.13%)	p<0.001
1-1.3 MPa	14805 (6.61%)	21950 (13.22%)	18366 (12.10%)	20044 (12.93%)	17131 (11.19%)	
1.3-1.6 MPa	7108 (3.17%)	32119 (19.35%)	35926 (23.67%)	26194 (16.90%)	30003 (19.60%)	
1.6-1.9 MPa	4901 (2.19%)	29658 (17.86%)	24649 (16.24%)	30442 (19.64%)	27501 (17.97%)	
1.9-2.2 MPa	2838 (1.27%)	17899 (10.78%)	16003 (10.54%)	15402 (9.94%)	27380 (17.89%)	
2.2-2.5 MPa	5852 (2.61%)	20827 (12.54%)	17980 (11.85%)	30599 (19.74%)	17442 (11.40%)	
2.5+ MPa	0 (0.00%)	25804 (15.54%)	22192 (14.62%)	14836 (9.57%)	15030 (9.82%)	

p - chi-squared test

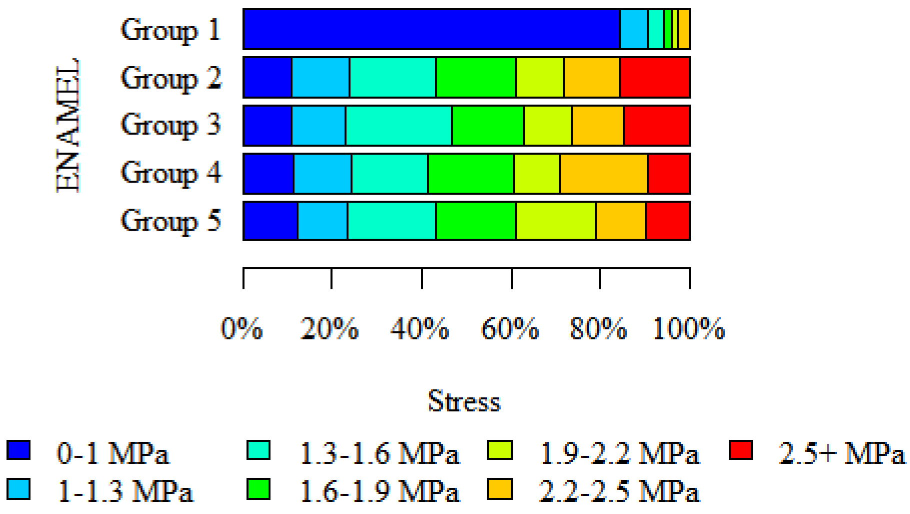

5.2 *MATERIAL: Filtek Z350 XT (3M ESPE, Seefeld, Germany)*

Stress	Group					p
	Group 1	Group 2	Group 3	Group 4	Group 5	
0-1 MPa	188104 (84.15%)	18535 (10.83%)	22168 (11.19%)	22303 (11.15%)	23159 (12.12%)	$p<0.001$
1-1.3 MPa	14517 (6.49%)	22884 (13.38%)	22829 (11.53%)	26016 (13.00%)	21575 (11.29%)	
1.3-1.6 MPa	6614 (2.96%)	33290 (19.46%)	48156 (24.32%)	34158 (17.07%)	37919 (19.84%)	
1.6-1.9 MPa	4835 (2.16%)	30688 (17.94%)	32376 (16.35%)	39137 (19.56%)	35088 (18.36%)	
1.9-2.2 MPa	3843 (1.72%)	17792 (10.40%)	18161 (9.17%)	39646 (19.81%)	34555 (18.08%)	
2.2-2.5 MPa	5633 (2.52%)	21132 (12.35%)	24800 (12.52%)	19069 (9.53%)	20515 (10.73%)	
2.5+ MPa	0 (0.00%)	26769 (15.65%)	29555 (14.92%)	19787 (9.89%)	18315 (9.58%)	

p - chi-squared test

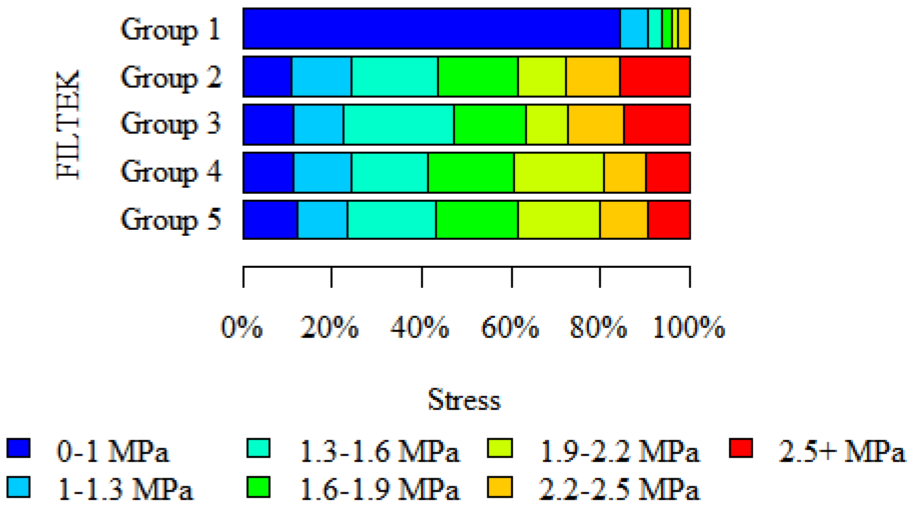

5.3 *MATERIAL: Grandio (VOCO, Cuxhaven, Germany)*

Stress	Group					p
	Group 1	Group 2	Group 3	Group 4	Group 5	
0-1 MPa	188103 (84.02%)	15654 (12.30%)	18261 (11.15%)	18747 (11.23%)	20052 (12.11%)	p<0.001
1-1.3 MPa	14315 (6.39%)	0 (0.00%)	19368 (11.82%)	22306 (13.36%)	19523 (11.79%)	
1.3-1.6 MPa	6931 (3.10%)	27471 (21.59%)	39487 (24.10%)	28214 (16.90%)	31210 (18.85%)	
1.6-1.9 MPa	4811 (2.15%)	25352 (19.92%)	26028 (15.89%)	33172 (19.87%)	31173 (18.83%)	
1.9-2.2 MPa	3891 (1.74%)	15577 (12.24%)	15761 (9.62%)	32330 (19.36%)	28253 (17.07%)	
2.2-2.5 MPa	5823 (2.60%)	18846 (14.81%)	20785 (12.69%)	15466 (9.26%)	18736 (11.32%)	
2.5+ MPa	0 (0.00%)	24353 (19.14%)	24130 (14.73%)	16741 (10.03%)	16582 (10.02%)	

p - chi-squared test

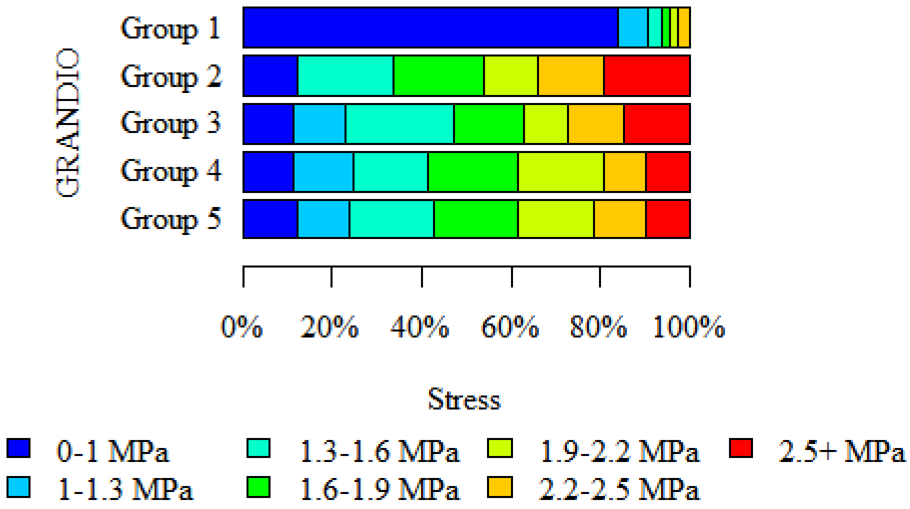

Supplement: Supplementary file 1 [file nanomaterials-10-01708-s001.pdf]
